# Physical activity and sleep problems in homeless adults

**DOI:** 10.1371/journal.pone.0218870

**Published:** 2019-07-05

**Authors:** Ashley Taylor, Rosenda Murillo, Michael S. Businelle, Tzu-An Chen, Darla E. Kendzor, Lorna H. McNeill, Lorraine R. Reitzel

**Affiliations:** 1 Department of Psychological, Health, and Learning Sciences, College of Education, The University of Houston, Houston, Texas, United States of America; 2 HEALTH Research Institute, University of Houston, Houston, Texas, United States of America; 3 School of Public Health, The University of Texas Health Science Center, Dallas, Texas, United States of America; 4 Department of Health Disparities Research, The University of Texas MD Anderson Cancer Center, Houston, Texas, United States of America; Centre for Addiction and Mental Health, CANADA

## Abstract

**Background:**

For the estimated 554,000 homeless individuals on any given night in the United States, obtaining quality sleep is often challenging. This group is known to have multiple health disparities, potentially affected by sleep problems; therefore, identifying lifestyle factors–such as physical activity–that are associated with improving both quality and quantity of sleep has important implications for public health. Here, we examine associations of physical activity with subjective sleep problems within a large sample of homeless adults.

**Methods:**

Participants were homeless adults recruited from Dallas and Oklahoma (N = 747; 66.1% men, M_age_ = 43.7±12.1). Participants self-reported insufficient sleep (number of days without sufficient rest/sleep in the last month; categorized as 0, 1–13, 14–29, or ≥30 days), sleep duration (over average 24 hours; categorized as ≤6 [short sleeper], 7–9 [optimal sleeper], or ≥10 hours [long sleeper]), and unintentional daytime sleep (number of days with unintentional sleep in the last month; categorized as 0 vs ≥30 days). Physical activity was assessed subjectively using the BRFSS Physical Activity Questionnaire. Regression analyses were performed to examine the associations between physical activity and sleep problems, controlling for age, sex, race, education, body mass, months homeless, at-risk drinking, self-rated health, serious mental illness, smoking status, and recruitment city.

**Results:**

Failure to meet/exceed physical activity guidelines was associated with higher likelihood of being a long sleeper (OR = 2.64, 95% CI: 1.46, 4.78) but a lower likelihood of having ≥30 days of insufficient rest/sleep (OR = 0.52, 95% CI: 0.29, 0.93).

**Conclusions:**

Findings suggest that physical activity promotion may hold promise for addressing the problem of too much sleep, but not other manifestations of sleep problems among this vulnerable group.

## Introduction

According to the National Sleep Foundation, it is recommended that younger adults (18–25) and adults (26–64) get between 7 and 9 hours of sleep each day [1]. For the estimated 554,000 individuals experiencing homelessness on any given night in the United States, the ability to obtain quality sleep (e.g., recommended sleep duration) is often challenging [2]. Problems with sleep can include subjective perceptions that the rest/sleep achieved was insufficient, sleep quantity/duration that is too short (≤6 hours) or too long (≥10 hours) [3], and episodes of unintentional daytime sleeping. Each of these manifestations of what is hereafter referred to as “sleep problems” may or may not be consistent with a sleep disorder [4]. Sleep is a vital component in human functioning, with the potential to affect both mental and physical health [5]. Research has suggested that sleep problems may be linked to negative health outcomes, affecting mood, cognition, energy, and physiological function [6]. For example, literature has supported a relationship between short sleep duration and increased risk of developing diabetes, hypertension, asthma, and arthritis [7].

Given the negative health outcomes related to sleep problems, identifying lifestyle factors associated with improving the quality and quantity of sleep among vulnerable populations has important implications for public health. Decades of epidemiologic research supports physical activity as one lifestyle factor linked to an abundance of health benefits, such as decreased risk for obesity and obesity related cancers [8], stronger cardiovascular health [9], and an overall improvement in both mental and physical health [10]. Likewise, the impact of daytime physical activity has been shown to stimulate greater periods of slow-wave sleep, known to be the deepest and most restorative stages of sleep [11]. Research among domiciled groups suggests that meeting national guideline recommendations of at least 150 minutes of physical activity per week can dramatically reduce several health-related concerns, including sleep problems [12]. Additionally, both acute and regular physical activity have been linked with small beneficial effects on total sleep duration and sleep efficiency, and those who get the recommended amount of physical activity have reported sleeping significantly better with increased alertness during the day [13,14]. Associations between physical activity and sleep problems have largely been studied among the general (domiciled) population, however, with limited work conducted among homeless individuals.

Evidence supports that both sleep quality and quantity can be environmentally influenced [15–17]; a fact of particular relevance to individuals who are homeless. Homeless individuals may face significant challenges in achieving adequate sleep due to harsh environmental conditions (e.g., extreme hot or cold weather), especially for those sleeping in open, public areas. One study conducted among homeless individuals found reports of increased adverse health outcomes when exposed to cold, wet winters as opposed to extreme heat, with greater reports of depression and inability to maintain overall wellness [18]. Additionally, individuals may lose sleep to remain vigilant, guarding against theft or assault when sleeping rough (i.e., on the street). Furthermore, even sheltered individuals may encounter significant sleep disruptions due to group sleeping accommodations, which often consist of cots placed alongside one another in large open areas. Reports from homeless drug users who stayed in emergency hostels and shelters cited lack of privacy, sleep disturbances, and uncomfortable sleeping conditions as difficulties experienced in attaining sleep [19]. Moreover, when compared to a control sample, women living in transitional housing were more likely to have higher subjective and objective sleep disturbances [20] and an additional study found a positive association between infrequent housing and insufficient sleep [3]. Because sleep problems may be affected by multiple and potentially unique challenges rendered by conditions of homelessness, better understanding modifiable factors associated with sleep insufficiency, short or long sleep duration, and unintentional sleep—such as physical activity—can provide inroads to eventual intervention development.

The purpose of this study was to gain a greater understanding of the association between physical activity and sleep problems among adults who are homeless. Although research supports that about 30% of homeless individuals do not achieve recommended levels of physical activity [21] and that sleep problems are prevalent within homeless samples [22,23], little to no research currently exists on associations between the two variables within this vulnerable group. As individuals who are homeless are known to have multiple health disparities that could be affected by sleep quality [24,25], better understanding physical activity and sleep among this vulnerable group is important for intervention planning and targeting. We hypothesized that meeting/exceeding physical activity guidelines would be associated with lower odds of subjective sleep problems within a large sample of homeless adults.

## Methods

### Participants

Participants comprised homeless adults recruited from two independent studies focused on health and health risk factors. The recruitment site for Study 1 was a single shelter in Dallas, TX. The recruitment sites for Study 2 were 6 agencies providing shelter and/or services to individuals in Oklahoma City, Oklahoma. In both studies, participants were recruited using flyers posted in the targeted settings and individuals were screened on site. Inclusion criteria required that participants be adults aged 18 or over, currently receiving services (e.g., shelter, food, counseling) at the targeted agencies, currently homeless (see [26] for more information on definition), English-speaking given that the questionnaires were provided only in English, and could demonstrate at least a 7^th^ grade English literacy level as indicated by a score of ≥4 on the Rapid Estimate of Adult Literacy in Medicine-Short Form [27]. Overall, 394 (Study 1) and 610 (Study 2) participants enrolled; however, only 244 enrollees in Study 1 (who were all homeless by virtue of residing at the shelter) were administered the sleep items and only 525 individuals from Study 2 endorsed current homelessness (i.e., self-identified as homeless and endorsed staying at a friend or family member’s house, homeless shelter, outside or on the street, hotel/motel, drug/alcohol treatment center, or other temporary location). The samples were further reduced to 234 (Study 1) and 513 (Study 2) based on missing data. More information on the sampling frame, including those excluded from participation, are referenced in other work [21–23,26].

### Procedures

Data collection took place at each recruitment site following the provision of informed consent. Enrolled participants completed questionnaires that were administered via laptop computer or tablet whereby survey items were visible on the screen and read aloud to the participant via headphones. Each participant received a $20 department store gift card as compensation for participating in the study. Data from Study 1 were collected in August 2013 and data from Study 2 were collected in July and August of 2016.

### Human subjects statement

Study 1: The Institutional Review Boards at The University of Texas Health Science Center, The University of Texas MD Anderson Cancer Center, and the University of Houston approved this study. Written informed consent for all study procedures was obtained before data collection.

Study 2: The Institutional Review Boards at the University of Oklahoma Health Sciences Center and the University of Houston approved this study. Informed consent for all study procedures was obtained before data collection.

### Measures

#### Participants’ characteristics

Participants were asked to self-report characteristics including lifetime homelessness (in months), which was included as a covariate in the current analysis based on known associations with sleep problems [28]. Additional participant characteristics included age, sex (reference group = male), race (reference group = minority), and educational attainment.

#### Mental and physical health

Participants self-reported a history of serious mental illness and self-rated health. Serious mental illness was assessed via self-report of any diagnosis of bipolar disorder, schizophrenia or schizoaffective disorder, or major depression over a lifetime. Self-reported history of serious mental illness (reference group = no mental illness) was included as a covariate in the current analysis based on known associations with circadian rhythm disturbance, insomnia, and hypersomnia [29–31]. Self-rated health was assessed with an item asking participants to rate their health in general, which was coded as excellent/very good/good (reference group) vs fair/poor. Self-rated health is well known as a predictor of morbidities that could impact sleep and physical activity and was included as a covariate in the current analysis [32–34]. The binary criterion variable was created and designed to focus on the correlates pertaining to suboptimal health, a common approach found within the literature [35].

#### Health behaviors

Participant smoking status (smoker or non-smoker) and at-risk drinking status (at-risk drinker or not at-risk drinker) were collected. Smoking status was assessed with 2 items: “Have you smoked at least 100 cigarettes in your lifetime?” and “Currently, do you smoke cigarettes on some days or every day?” Participants were considered smokers if they answered yes to both items. Smoking status (reference group = non-smoker) was included as a covariate in the current analysis based on known associations with difficulty initiating sleep and sleep fragmentation [36]. Alcohol use was assessed using the Alcohol Quantity and Frequency Questionnaire [37], which assessed average alcohol consumption on each day of the week over the last 30 days. Participants were shown visual images of what constituted 1 standard drink (i.e., 4-5-ounce glass of wine, shot of liquor, 12-ounce beer). The at-risk drinking variable was defined as consuming >14 drinks per week for men and >7 drinks per week for women OR >5 drinks in a drinking episode in the past 30 days for men and >4 drinks for women. At-risk drinking status (reference group = not at-risk drinker) was included as a covariate in the current analysis based on known associations with insomnia [38]. Finally, participants’ body mass index (BMI) was assessed using a stadiometer and scale, calculated from height and weight (kg/m^2^), and categorized as underweight/normal weight: BMI <25 (reference group), overweight: BMI 25–30, or obese: BMI >30). Of note, only 1.88% (n = 14) participants were underweight in this sample; thus, they were combined with normal weight participants to avoid model convergence issues.

#### Physical activity

Physical activity was assessed subjectively using the Behavioral Risk Factor Surveillance System (BRFSS) Physical Activity Questionnaire [39]. Participants reported the quantity (>10 minutes in duration) and frequency (days of the week) of moderate and vigorous non-employment related physical activity over the previous week. Moderate physical activity was characterized by anything causing an increase in breathing or heart rate (e.g., brisk walking) and vigorous activity was characterized by anything causing a significant increase in breathing or heart rate (e.g., running). From these self-reports, the total physical activity in minutes per week was calculated, which was equal to (vigorous mins *2) + (moderate mins) [12]. Total physical activity was then classified as not meeting recommendations (<150 min/week) versus meeting/exceeding recommendations (≥150 min/week [12]; reference group) as the primary predictor variable. For further exploration, it was additionally classified as: (1) 0 min/week = inactive, (2) 1 to 149 min/week = insufficiently active, (3) 150 to 299 min/week = sufficiently active, or (4) ≥300 min/week = highly active.

#### Sleep problems

Sleep problems were assessed subjectively using individual sleep items from the Behavioral Risk Factor Surveillance System (BRFSS) [40]. Participants reported: (1) the number of days over the past month in which they did not get enough rest or sleep (insufficient sleep); (2) the average number of hours of sleep they typically obtain in a 24-hour period (sleep duration); and (3) the number of days over the past month in which they found themselves unintentionally falling asleep during the day (unintentional sleep). Categorized sleep variables were as follows: (1a) insufficient sleep: 0 days (reference group), 1–13 days, 14–29 days, and ≥30 days [41]; (2a) sleep duration: ≤6 hours (short sleeper; reference group), 7–9 hours (optimal sleeper), ≥10 hours (long sleeper) [3]; and (3a) unintentional sleep: 0 days (reference group) versus ≥1 day [42]. Categorized variables were the primary criterion variables based on their use in prior literature to facilitate comparison [3,41,42], with continuous variables used to provide descriptive information.

### Data analysis

Within sample comparisons based on physical activity guideline adherence were performed using ANOVA or Chi-square tests for continuous and categorical variables, respectively. To assess the relation between physical activity and sleep problems, multinomial logistic regressions and binary logistic regression analyses were performed depending on the nature of the sleep variables. Multinomial logistic regressions were conducted when the outcome variables were days of insufficient rest/sleep and sleep duration. Binary logistic regression analysis was performed for unintentional sleep. The covariates include age, sex, race, years of education, BMI, months homeless over the lifetime, at-risk drinking status, self-rated health, serious mental illness, smoking status, and recruitment city. Odds ratios (ORs) and 95% confidence intervals (CIs) were calculated for the sleep variables. All analyses were conducted using SAS [43]. Level of significance was designated at *p* < .05.

## Results

### Participants’ characteristics

The overall sample (N = 747; 68.7% recruited in Oklahoma) comprised 66.1% men (n = 494) and 52.8% of all participants (n = 394) self-identified as being of a racial/ethnic minority group (i.e., 34.7% Black or African American, 0.7% Asian, 0.5% Native Hawaiian or Pacific Islander, 8.4% American Indian/Alaska Native, 7.8% >one race, 0.7% other). See Table 1 for participants’ characteristics overall and by physical activity guideline adherence.

**Table 1:** Participants’ characteristics by physical activity (PA) guideline adherence (N = 747).

	Total (N = 747)	Met/Exceeded PA Guidelines (N = 447)	Did Not Meet PA Guidelines (N = 300)		
*Variables of Interest*	M (SD) / % [N]	M (SD) / % [N]	M (SD) / % [N]	Statistic	p value
Sex				5.8934^╪^	0.0152
Male	66.13 [494]	69.57 [311]	61.00 [183]		
Female	33.87 [253]	30.43 [136]	39.00 [117]		
Race				0.3734^╪^	0.5412
White	47.18 [352]	48.10 [215]	45.82 [137]		
Minority	52.82 [394]	51.90 [232]	54.18 [162]		
Age	43.73 (12.07)	43.54 (12.36)	44.02 (11.65)	0.29^┼^	0.5922
Educational attainment (in years)	12.00 (2.00)	12.08 (1.97)	11.88 (2.05)	1.8^┼^	0.1802
Lifetime months homeless	43.71 (51.71)	43.31 (50.95)	44.3 (52.91)	0.06^┼^	0.7996
BMI				8.6052^╪^	0.0135
Underweight/Normal weight	38.61 [288]	41.93 [187]	33.67 [101]		
Overweight	29.22 [218]	29.82 [133]	28.33 [85]		
Obese	32.17 [240]	28.25 [126]	38.00 [114]		
Self-rated health				17.1346^╪^	<0.0001
Excellent, very good, or good	66.40 [496]	72.26 [323]	57.67 [173]		
Fair or poor	33.60 [251]	27.74 [124]	42.33 [127]		
Serious mental illness				0.3592^╪^	0.5489
No	34.27 [256]	35.12 [157]	33.00 [99]		
Yes	65.73 [491]	64.88 [290]	67.00 [201]		
At-risk drinker				1.1258^╪^	0.2887
No	63.72 [476]	62.19 [278]	66.00 [198]		
Yes	36.28 [271]	37.81 [169]	34.00 [102]		
Smoker				0.0053^╪^	0.9421
No	19.44 [139]	19.53 [83]	19.31 [56]		
Yes	80.56 [576]	80.47 [342]	80.69 [234]		
Recruitment city				26.4756^╪^	<0.0001
Dallas	31.33 [234]	38.48 [172]	20.67 [62]		
Oklahoma	68.67 [513]	61.52 [275]	79.33 [238]		
Total weekly physical activity (minutes)	837.89 (1492.74)	1381.27 (1728.95)	28.25 (38.42)	183.58^┼^	<0.0001
Non-employment related physical activity				747^╪^	<0.0001
Inactive (0 min/week)	19.68 [147]	0.00 [0]	49.00 [147]		
Insufficiently active (1 to 149 min/week)	20.48 [153]	0.00 [0]	51.00 [153]		
Sufficiently active (150 to 299 min/week)	10.84 [81]	18.12 [81]	0.00 [0]		
Highly active (≥ 300 min/week)	49.00 [366]	81.88 [366]	0.00 [0]		
Days of insufficient rest/sleep	12.51 (11.21)	12.89 (11.26)	11.96 (11.13)	1.22^┼^	0.2691
Days of insufficient rest/sleep				2.715^╪^	0.4377
0 days	14.00 [104]	12.61 [56]	16.05 [48]		
1–13 days	45.22 [336]	45.05 [200]	45.48 [136]		
14–29 days	23.01 [171]	23.20 [103]	22.74 [68]		
≥ 30 days	17.77 [132]	19.14 [85]	15.72 [47]		
Sleep duration (in hours)	6.73 (2.17)	6.60 (2.03)	6.92 (2.36)	3.91^┼^	0.0484
Sleep duration				8.3372^╪^	0.0155
≦ 6 hours (short sleeper)	49.59 [366]	51.47 [227]	46.80 [139]		
7–9 hours (optimal sleeper)	41.87 [309]	42.40 [187]	41.08 [122]		
≧ 10 hours (long sleeper)	8.54 [63]	6.12 [27]	12.12 [36]		
Days with unintentional sleep	5.15 (8.18)	5.56 (8.64)	4.54 (7.42)	2.82^┼^	0.0933
Days with unintentional sleep				0.4976^╪^	0.4806
0 days	36.81 [275]	35.79 [160]	38.33 [115]		
≧ 1 day	63.19 [472]	64.21 [287]	61.67 [185]		

*Note*: Within sample comparisons based on physical activity guideline adherence were performed using ANOVA or Chi-square tests for continuous and categorical variables, respectively

^┼^ANOVA

^╪^Chi-square test.

Relative to those not meeting physical activity guidelines, participants meeting/exceeding physical activity guidelines were significantly more likely to be men versus women (69.6% vs 61.0%), report excellent, very good or good versus fair/poor health (72.3% vs 57.7%) and be from the Dallas versus Oklahoma City sample (79.3% vs 61.5%). Those meeting/exceeding physical activity guidelines had the highest proportion of individuals who reported underweight/normal weight (41.9%), while those not meeting physical activity guidelines had the highest proportion of individuals who were obese (38.0%). Those meeting/exceeding physical activity guidelines were more likely to report fewer hours of sleep (6.6 vs 6.9) and had fewer long sleepers (≥10hrs; 6.1% vs 12.1%) relative to participants who did not meet physical activity guidelines.

Correlations between the sleep problem variables and covariates are presented in Table 2. More days of insufficient rest/sleep were associated with women and white (vs minority race) participants. Positive correlations were found between insufficient rest/sleep and fair/poor self-rated health and serious mental illness. Longer sleep duration was associated with minority (vs white race) participants, excellent/very good/good self-rated health, not being an at-risk drinker, and fewer days of insufficient rest/sleep. More days with unintentional sleep were associated with being of minority (vs white) race, lower educational attainment, and more days of insufficient rest/sleep.

**Table 2:** Intercorrelations of participants’ characteristics and sleep variables among a homeless sample (N = 747).

	1	2	3	4	5	6	7	8	9	10	11	12	13
1. Sex^a^	1	0.046	-0.127***	0.049	-0.123***	0.158***	0.088*	0.162***	-0.134***	-0.036	0.081*	0.054	-0.029
2. Race^b^		1	0.029	-0.080*	-0.043	-0.018	0.146***	0.100**	-0.120**	0.074*	0.116***	-0.076*	-0.131***
3. Age			1	0.105**	0.242***	0.025	0.113**	0.021	0.061	-0.031	-0.049	-0.012	-0.030
4. Educational attainment				1	-0.070	0.078*	-0.052	-0.007	-0.055	-0.128***	-0.013	-0.024	-0.073*
5. Lifetime months homeless					1	-0.041	0.061	0.019	0.110**	0.042	0.037	0.029	0.036
6. BMI^c^						1	0.067	0.059	-0.136***	-0.141***	-0.048	-0.044	-0.035
7. Self-rated health^d^							1	0.119**	-0.003	0.051	0.185***	-0.121***	0.050
8. Serious mental illness^e^								1	0.029	0.026	0.181***	-0.046	0.046
9. At-risk drinker^f^									1	0.046	0.049	-0.127***	0.040
10. Smoker^g^										1	0.067	-0.025	-0.042
11. Days of insufficient rest/sleep^h^											1	-0.361***	0.185***
12. Sleep duration^i^												1	0.019
13. Days with unintentional sleep^j^													1

*Note*: Associations between continuous variables were assessed using Pearson product moment correlations, associations between categorical variables and continuous variables were assessed using Point-biserial correlations, and associations between categorical variables were assessed using Phi coefficients.

*p<0.05

**p<0.01

***p<0.001.

^a^1 = Male; 2 = Female.

^b^1 = Minority; 2 = White.

^c^1 = Underweight/Normal weight; 2 = Overweight; 3 = Obese.

^d^1 = Excellent/very good/good; 2 = Fair/poor.

^e^1 = No serious mental illness; 2 = Serious mental illness.

^f^1 = Not at-risk drinker; 2 = At-risk-drinker.

^g^1 = Non-smoker; 2 = Smoker.

^h^1 = 0 days insufficient rest/sleep; 2 = 1–13 days insufficient rest/sleep; 3 = 14–29 days insufficient rest/sleep; 4 = ≥30 days insufficient rest/sleep.

^i^1 = ≤ 6 hours; 2 = 7–9 hours; 3 = ≥ 10 hours.

^j^1 = 0 days with unintentional sleep; 2 = ≥1 day with unintentional sleep.

#### Relation between days of insufficient rest/sleep and physical activity

The adjusted multinomial logistic regression showed that the odds ratio of changing from meeting/exceeding physical activity guidelines to not meeting guidelines was 0.522 (OR: 0.292–0.932) for insufficient rest/sleep ≥ 30 days versus 0 days. That is, the expected risk of insufficient sleep for 30 days or more was significantly lower for participants not meeting physical activity guidelines than meeting/exceeding guidelines (Table 3). Further exploration with a 4-level physical activity variable (inactive, insufficiently active, sufficiently active, or highly active) showed that the odds ratio of changing from being highly active (≥ 300 min/week) to insufficiently active (1–149 min/week) was 0.394 (OR: 0.184–0.844) for insufficient rest/sleep (≥ 30 days) (data not shown in Table). In other words, the likelihood of reporting insufficient sleep for 30 days or more was greater among those who were highly active relative to those who were insufficiently active.

**Table 3:** Relation between days of insufficient rest/sleep and meeting/exceeding physical activity guidelines (N = 747).

Sleep Category	1–13 days		14–29 days		≥ 30 days	
	B (SE)	OR (95% CI)	B (SE)	OR (95% CI)	B (SE)	OR (95% CI)
Sex^a^	0.0911 (0.2698)	1.095 (0.645, 1.859)	0.3638 (0.2984)	1.439 (0.802, 2.582)	0.2427 (0.3098)	1.275 (0.695, 2.339)
Race^b^	-0.2232 (0.2527)	0.8 (0.487, 1.313)	0.1383 (0.2846)	1.148 (0.657, 2.006)	0.202 (0.2996)	1.224 (0.68, 2.202)
Age	0.0104 (0.0197)	0.999 (0.978, 1.019)	0.0118 (0.5771)	0.991 (0.969, 1.014)	0.0123 (3.1206)	0.978 (0.955, 1.002)
Educational attainment	0.014 (0.0562)	1.014 (0.908, 1.132)	0.0491 (0.0662)	1.05 (0.922, 1.196)	0.015 (0.0675)	1.015 (0.889, 1.159)
Lifetime months homeless	0.00595 (0.00306)	1.006 (1, 1.012)	0.00653 (0.00326)	1.007 (1, 1.013)	0.00512 (0.00348)	1.005 (0.998, 1.012)
BMI (Obese vs Underweight/Normal)^c^	-0.3248 (0.2978)	0.723 (0.403, 1.295)	-0.7662 (0.3397)	0.465 (0.239, 0.904)	-0.5234 (0.3526)	0.593 (0.297, 1.183)
BMI (Overweight vs Underweight/Normal)^c^	-0.3565 (0.3007)	0.7 (0.388, 1.262)	-0.3222 (0.3297)	0.725 (0.38, 1.383)	-0.3322 (0.3492)	0.717 (0.362, 1.422)
Self-rated health^d^	0.4308 (0.2865)	1.538 (0.877, 2.698)	0.9658 (0.3097)	2.627 (1.432, 4.82)	1.1998 (0.3193)	3.32 (1.776, 6.206)
Serious mental illness^e^	0.3411 (0.2451)	1.406 (0.87, 2.274)	0.8108 (0.286)	2.25 (1.284, 3.941)	1.0284 (0.3085)	2.796 (1.528, 5.119)
At-risk drinker^f^	0.0997 (0.2645)	1.105 (0.658, 1.855)	0.5302 (0.2916)	1.699 (0.96, 3.009)	0.1356 (0.3099)	1.145 (0.624, 2.102)
Smoker^g^	0.0735 (0.2866)	1.076 (0.614, 1.888)	0.3486 (0.3402)	1.417 (0.727, 2.76)	0.3114 (0.3538)	1.365 (0.682, 2.732)
Recruitment city^h^	0.7101 (0.3154)	2.034 (1.096, 3.774)	0.8577 (0.3469)	2.358 (1.195, 4.653)	0.0232 (0.3827)	1.023 (0.483, 2.167)
Met/Exceeded physical activity guidelines^i^	-0.2246 (0.2475)	0.799 (0.492, 1.298)	-0.2629 (0.2794)	0.769 (0.445, 1.329)	-0.6503 (0.2961)	0.522 (0.292, 0.932)

*Note*. Analysis was conducted using adjusted multinomial logistic regression. Insufficient Rest/Sleep: reference group = 0 days.

^a^1 = Male (reference group); 2 = Female.

^b^1 = Minority (reference group); 2 = White.

^c^1 = Underweight/Normal weight (reference group); 2 = Overweight; 3 = Obese.

^d^ 1 = Excellent/very good/good (reference group); 2 = Fair/poor.

^e^1 = No serious mental illness (reference group); 2 = Serious mental illness.

^f^ 1 = Not at-risk drinker (reference group); 2 = At-risk-drinker.

^g^1 = Non-smoker (reference group); 2 = Smoker.

^h^1 = Oklahoma (reference group); 2 = Dallas.

^i^1 = Met/exceeded physical activity guidelines (reference group); 2 = Did not meet physical activity guidelines.

#### Relation between sleep duration and physical activity

The odds ratio of changing from meeting/exceeding physical activity guidelines to not meeting guidelines was 2.643 (OR: 1.463–4.776) for being a long sleeper (≥10 hours) versus short sleeper (≤6 hours). That is, the expected risk of being a long sleeper was significantly higher for participants who did not meet the physical activity guidelines relative to those who met/exceeded them (Table 4). Further exploration with a 4-level physical activity variable indicated that the odds ratios of changing from being highly active (≥300 min/week) to insufficiently active (1–149 min/week) and inactive (0 min/week) was 2.569 (OR: 1.174–5.62) and 3.378 (OR: 1.624–7.028) for being a long sleeper relative to short sleeper, respectively (data not shown in Table). In other words, the expected odds of being a long sleeper was significantly lower for those who were among the most highly active in the sample.

**Table 4:** Relation between sleep duration and meeting/exceeding the physical activity guidelines (N = 747).

Sleep Category	Optimal Sleeper		Long Sleeper	
	B (SE)	OR (95% CI)	B (SE)	OR (95% CI)
Sex^a^	0.0704 (0.1824)	1.073 (0.75, 1.534)	0.8159 (0.3094)	2.261 (1.233, 4.147)
Race^b^	-0.2783 (0.1732)	0.757 (0.539, 1.063)	-0.2932 (0.3086)	0.746 (0.407, 1.366)
Age	0.011 (0.00712)	1.011 (0.997, 1.025)	-0.00666 (0.0129)	0.993 (0.969, 1.019)
Educational attainment	-0.0201 (0.0411)	0.98 (0.904, 1.062)	-0.0516 (0.0703)	0.95 (0.827, 1.09)
Lifetime months homeless	0.0015 (0.00163)	1.002 (0.998, 1.005)	0.00379 (0.00283)	1.004 (0.998, 1.009)
BMI (Obese vs Underweight/Normal weight)^c^	-0.2662 (0.2019)	0.766 (0.516, 1.138)	-0.4786 (0.3519)	0.62 (0.311, 1.235)
BMI (Overweight vs Underweight/Normal weight)^c^	0.0127 (0.1996)	1.013 (0.685, 1.498)	-0.3771 (0.3633)	0.686 (0.336, 1.398)
Self-rated health^d^	-0.3401 (0.1771)	0.712 (0.503, 1.007)	-1.1321 (0.3595)	0.322 (0.159, 0.652)
Serious mental illness^e^	-0.1285 (0.1769)	0.879 (0.622, 1.244)	-0.2796 (0.3085)	0.756 (0.413, 1.384)
At-risk drinking^f^	-0.5352 (0.1767)	0.586 (0.414, 0.828)	-0.6726 (0.3303)	0.51 (0.267, 0.975)
Smoker^g^	-0.1011 (0.209)	0.904 (0.6, 1.361)	-0.1251 (0.3653)	0.882 (0.431, 1.805)
Recruitment city^h^	0.3036 (0.1964)	1.355 (0.922, 1.991)	0.2284 (0.3483)	1.257 (0.635, 2.487)
Met/exceeded physical activity guidelines^i^	0.1618 (0.1724)	1.176 (0.839, 1.648)	0.9719 (0.3019)	2.643 (1.463, 4.776)

*Note*: Analysis was conducted using adjusted multinomial logistic regression. Sleep duration: reference group = short sleeper (≤6hrs).

^a^1 = Male (reference group); 2 = Female.

^b^1 = Minority (reference group); 2 = White.

^c^1 = Underweight/Normal weight (reference group); 2 = Overweight; 3 = Obese.

^d^ 1 = Excellent/very good/good (reference group); 2 = Fair/poor.

^e^1 = No serious mental illness (reference group); 2 = Serious mental illness.

^f^ 1 = Not at-risk drinker (reference group); 2 = At-risk-drinker.

^g^1 = Non-smoker (reference group); 2 = Smoker.

^h^1 = Oklahoma (reference group); 2 = Dallas.

^i^1 = Met/exceeded physical activity guidelines (reference group); 2 = Did not meet physical activity guidelines.

#### Relation between unintentional sleep and physical activity

The association between meeting/exceeding physical activity guidelines and unintentional sleep was not significant, nor were associations between a 4-level physical activity variable and unintentional sleep.

## Discussion

Homeless adults are known to have multiple health disparities, potentially affected by sleep problems [25]; therefore, identifying lifestyle factors–such as physical activity–that could be associated with better quality and quantity of sleep is important. This study was the first, to the authors’ knowledge, to explore the association between physical activity and subjective sleep problems among a large sample of homeless adults in the United States. Results of this study indicated that meeting/exceeding physical activity guidelines was associated with a greater likelihood of pervasive sleep insufficiency (insufficient sleep experienced ≥30 days a month), and a lower likelihood of long sleep duration. Meeting physical activity guidelines had no association with unintentional daytime sleep. Thus, with the exception of avoiding long sleep duration, the positive association between physical activity and sleep quality found among domiciled samples were not replicated in this sample of adults who were homeless [11,44]. Nevertheless, results are valuable in that long sleep duration has known associations with negative health outcomes, including increased risk of developing diabetes, hypertension, and CVD [45,46], as well as higher rates of premature mortality [47]. Although not directly measured in this study, these results suggest that interventions to increase physical activity may hold promise for addressing long sleep duration among adults who are homeless, as routine physical activity has been known to regulate sleep cycles to promote sleep efficiency with the potential to mitigate both long and short sleep duration [48]. However, it is important to note that of the 300 participants in our study who reported not meeting physical activity guidelines, only 12.1% reported being long sleepers. Moreover, the actual difference in the number of long sleepers who met physical activity guidelines (n = 27) versus those who did not (n = 36) was rather small. Future work should explore these associations among other homeless samples, and particularly those living in less temperate climates where physical activity and sleep may be affected by more varying climates and precipitation rates.

Results reveal distinctive physical activity and sleep problem characteristics among this homeless sample as compared with known data on domiciled samples. For example, more than half of our sample (59.8%) met or exceeded weekly physical activity guidelines relative to 21% as reported within a large nationally representative sample of domiciled adults [49]. Likewise, our sample had a lower proportion of individuals endorsing no physical activity (19.7%) relative to that reported by domiciled adults in Texas (27.2%) and Oklahoma (31.2%) [50]. With regard to sleep, a lower proportion of our sample reported zero days of insufficient rest/sleep (14.0%) relative to what has been reported in domiciled samples (31.0%) and a slightly higher proportion of our sample (17.8%) reported ≥30 days of insufficient rest/sleep relative to a domiciled sample (11.1%) [41]. More than half of the participants in the current study did not obtain the recommended 7 to 9 hours of sleep over a 24-hour period [1]. In addition, a greater proportion of our sample reported ≤6 hours sleep (49.6% vs 31.1%) and ≥10 hours sleep (8.5% vs 4.1%) as compared with a large nationally representative sample of adults aged 45 or older [3]. Furthermore, reports of at least one day with unintentional sleep were greater (63.2%) among our sample relative to a national domiciled sample (37.9%) [42].

Although not measured in this study, there are several reasons why homeless individuals may report more physical activity than their domiciled counterparts. First, while all day homeless shelters exist, they are not widely available; this forces these individuals to occupy the streets, where they must walk to a store or restaurant to procure goods, as many do not have access to personal transportation [51]. This lack of access to personal transportation may lend to an increase in physical activity. Furthermore, homeless individuals are particularly vulnerable to criminal victimization and elevated risk of being confronted by the police for violations of minor laws; moving from place to place may help avoid such issues [52,53]. Likewise, although not measured directly, reasons for poor sleep in this vulnerable group may mirror those contributing to high physical activity including safety concerns, victimization vulnerability, etc [22]. Future qualitative studies in this area may better delineate the veracity of these suppositions. The prevalence of sleep problems in homeless adults, however, coupled with the known negative effects of poor sleep on health [3,22,23,42], suggest the importance of future work in addressing problems with sleep among this group.

Overall, the largely null findings in this study regarding associations between physical activity and sleep were unexpected based on work conducted among domiciled adults. Even significant findings whereby meeting/exceeding physical activity guidelines was associated with a greater likelihood of pervasive sleep insufficiency (insufficiency experienced ≥30 days a month) was not in line with domiciled adult research [13]. Our data were insufficient to delineate the reason for this pattern of results; however, true differences between homeless adults versus domiciled adults (as described above) may have influenced them. Also, evidence suggests that physical activity may lose its positive effect on overall health, including sleep, with increased stress levels [54] and individuals who are homeless may have significantly more stressors than even low-income domiciled counterparts (cf. [55]). Consequently, physical activity may not have the same benefits on sleep among very high stress groups relative to lower stress segments of the population (i.e., domiciled adults). It is also possible that variables measured in this study or whose influence is not sufficiently controlled for by our covariates, may underlie the pattern of results described herein. Given the known benefits of physical activity on health [8–13], however, recommendations to decrease physical activity in the service of reducing pervasive sleep insufficiency would be misbegotten. Case in point, meeting/exceeding physical activity guidelines was associated with several positive things, including better perception of general health and healthy BMI, in this sample. Overall, however, more work is needed to see if this pattern of results generalizes to other samples of individuals who are homeless and to delineate possible reasons for the link between high physical activity and pervasive sleep insufficiency.

Several limitations exist within the current study. First, this sample was recruited from shelters located in the West South-Central region of the United States, therefore caution should be exercised in generalizing these findings to homeless adults living in other geographical locations. Despite this, the Dallas and Oklahoma City samples studied here were fairly representative of the non-domiciled populations in those cities [23,55]. Moreover, data from Study 1 could be considered dated, as it was collected in August 2013. Notwithstanding, results should be replicated with other samples, and future studies in this area might consider statistical adjustment for multiple comparisons. Another limitation was the inability to evaluate a causal relationship between physical activity and subjective sleep problems because the data were cross-sectional. Additionally, the cross-sectional nature of the study precludes better understanding the bidirectional relationship between physical activity and sleep [56]. The use of self-report measures to assess both the primary predictor and the outcome variable increased the likelihood of recall bias; thus, future studies should consider additional methods of measurement, including the use of actigraphy to increase the rigor of the scientific analysis. Finally, we were unable to account for the ways in which our sample were physically active or the reasons why they were or were not active. Qualitative data would better contextualize physical activity, as well as perceptions regarding the basis of sleep problems, in future work among this group.

In conclusion, findings from this study fill an important gap within the literature by shedding light on the association between physical activity and sleep problems within an adult homeless population. Results indicated that failure to meet/exceed physical activity guidelines was associated with sleeping too long; thus, interventions to promote physical activity among homeless samples may have the potential to mitigate negative health outcomes associated with this, but not other, manifestations of sleep problems.

## Supporting information

S1 Dataset PLoS_ONE_supporting_information_supplementary_dataset. (XLSX)
